# Thoracoscopic pneumonectomy for massive haemoptysis secondary to chronic pulmonary vein stenosis post-atrial fibrillation ablation

**DOI:** 10.1093/icvts/ivaf166

**Published:** 2025-07-22

**Authors:** Fumie Osuga, Kentaro Minegishi, Shunsuke Endo, Hiroyoshi Tsubochi

**Affiliations:** Department of General Thoracic Surgery, Jichi Medical University, Saitama Medical Center, Saitama-shi, Saitama 330-8503, Japan; Department of General Thoracic Surgery, Jichi Medical University, Saitama Medical Center, Saitama-shi, Saitama 330-8503, Japan; Department of General Thoracic Surgery, Jichi Medical University, Saitama Medical Center, Saitama-shi, Saitama 330-8503, Japan; Department of General Thoracic Surgery, Jichi Medical University, Saitama Medical Center, Saitama-shi, Saitama 330-8503, Japan

**Keywords:** haemoptysis, pulmonary vein stenosis, catheter ablation

## Abstract

Pulmonary vein (PV) stenosis is a rare but serious complication after transcatheter ablation for atrial fibrillation, potentially leading to massive haemoptysis. We present a case of severe PV stenosis resulting in haemoptysis. A 57-year-old man presented with haemoptysis 18 months after catheter ablation. His left PV was almost completely occluded and required the left pneumonectomy. Pathological examination revealed irreversible fibrosis of the PV intima, which led to pulmonary congestion. Surgical intervention resolved the haemoptysis. Anatomical pulmonary resection is required for massive haemoptysis caused by PV stenosis that occurs long after transcatheter ablation.

## INTRODUCTION

Transcatheter ablation is a common and effective therapeutic strategy for atrial fibrillation. Pulmonary vein (PV) stenosis is a rare complication of transcatheter ablation that can cause cough, dyspnoea, fatigue, and occasionally massive haemoptysis, though such severe cases are rarely reported. Severe PV stenosis caused by transcatheter ablation is sometimes irreversible and may require anatomical lung resection to control symptoms. Herein, we report a case treated with thoracoscopic left pneumonectomy for massive haemoptysis caused by severe PV stenosis following transcatheter ablation for atrial fibrillation.

## CASE

We affirm this research adheres to the ethical standards and guidelines set by Jichi Medical University Saitama Medical Center and has received approval from Institutional Review Board (IRB) on 9 October 2024. The IRB approval number is S24-070. Informed consent was obtained from the patients in writing.

**Figure 1.**
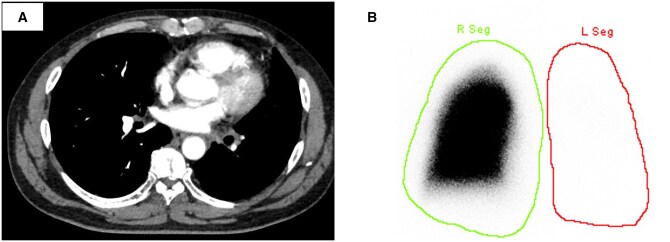
Computed tomography showed severe stenosis of the left pulmonary vein (A). Ventilation-perfusion scintigraphy showed little or no blood flow in the left lung (B).

A 57-year-old man underwent transcatheter ablation for atrial fibrillation 18 months ago at another hospital. Three months after the procedure, the patient developed a persistent cough and fatigue. A chest X-ray revealed no abnormalities, so he was observed without further imaging. He presented to the emergency department with sudden haemoptysis. Computed tomography (CT) showed severe left superior and inferior PV stenosis (**Figure 1A**). Ventilation-perfusion scintigraphy showed that the left lung perfusion was severely diminished, receiving only 2% of the blood flow (**Figure 1B**).

**Figure 2.**
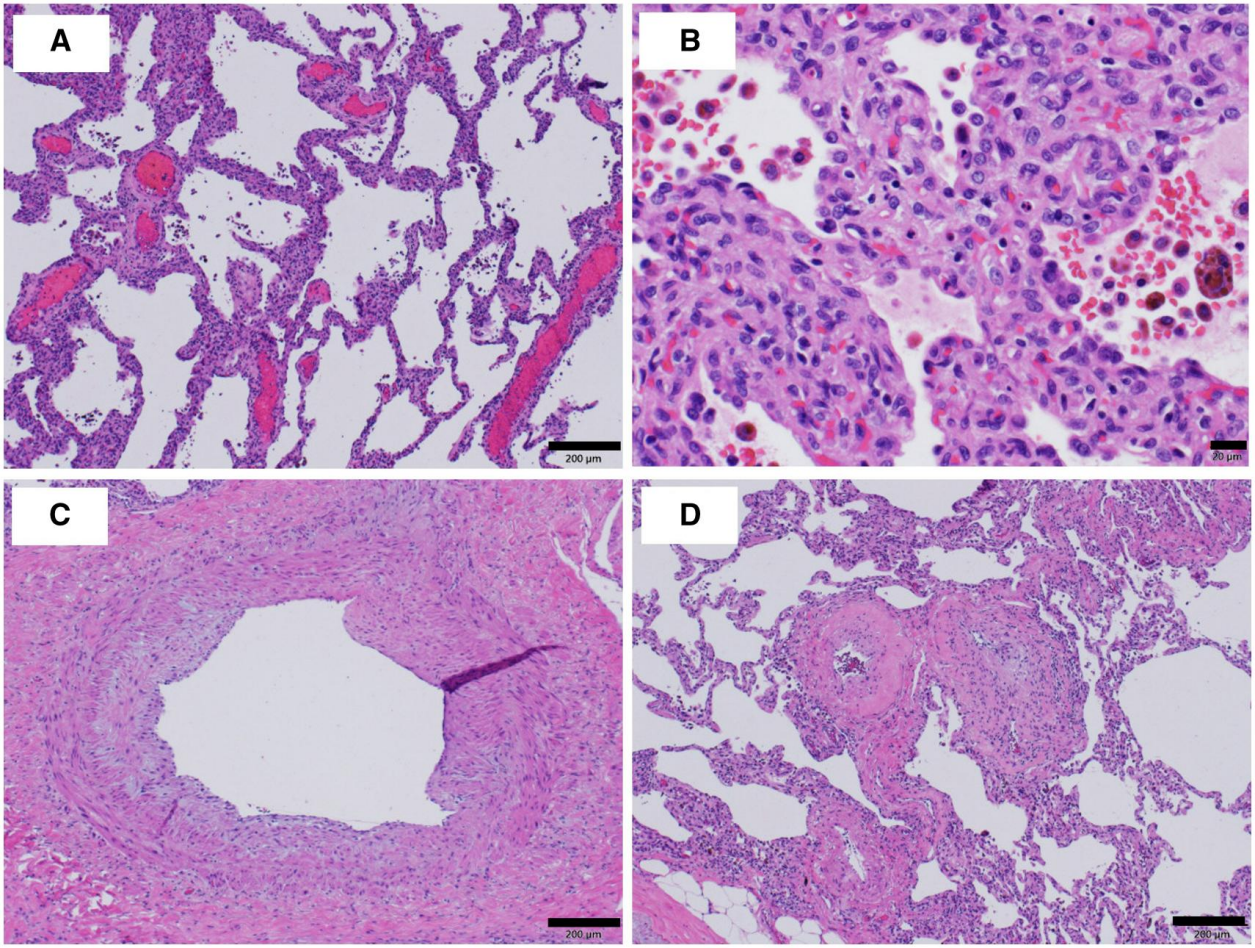
The pathology showed congestion (A), capillary hyperplasia, alveolar haemorrhage, and hemosiderin-laden macrophages (B). Fibrous thickening of the pulmonary vein wall was present both centrally (C) and peripherally (D). Some pulmonary veins were obstructed (C, D).

Thoracoscopic left pneumonectomy was performed. Operative findings showed enlarged PVs on the surface of the lung. Small vessels around the pulmonary ligament were also enlarged. The operation time was 288 minutes, and the blood loss was 327 mL. Microscopic examination of the lungs showed pulmonary congestion (**Figure 2A**), increased capillary growth, alveolar haemorrhage, and hemosiderin-laden macrophages (**Figure 2B**). The PVs from the centre (**Figure 2C**) to the capillaries (**Figure 2D**) had a narrowed lumen due to fibrous thickening of the intima. The bronchial arteries were not developed. The pleura showed dilation and enlargement of vessels, venous predominance, and lymphatic ducts. The preoperative PaO_2_/FiO_2_ ratio was 190, improving to 370 by the end of surgery. Symptoms improved postoperatively and were maintained for 1 year. During this period, we performed CT at 3 months, 6 months, and 1 year after surgery and confirmed that there were no abnormal findings.

## DISCUSSION

Transcatheter ablation is an established treatment for atrial fibrillation when medical therapy fails to control symptoms. One of the serious complications is PV stenosis. Previous reports have placed the incidence of PV stenosis at 0%-42%. A recent review reported a mean incidence of 2% and a median incidence of 3.1%.^1^ The degree of PV stenosis is classified as mild if less than 50%, moderate if 50%-69%, and severe if 70% or greater.^2^ The severity of symptoms of PV stenosis is thought to depend on the degree of stenosis, cough, and dyspnoea are common, but haemoptysis is rare. Treatment for PV stenosis includes balloon dilatation or stenting, but recurrence rates remain high: 30%-87% (mean 60%) for balloon dilatation and 14%-57% (mean 34%) for stent placement.^3^

In our case, microvascular sclerosis was already complete. If severe stenosis of the large PV persists over time, the small PVs and small pulmonary arteries are also exposed to high pressure and become stenotic, leading to pulmonary congestion and alveolar haemorrhage.

Severe PV stenosis reduces pulmonary artery blood flow, which can be evaluated by pulmonary perfusion scintigraphy. If perfusion is severely diminished, lung resection may improve V/Q mismatch and lung function. If the PV stenosis is single or unilateral, lung resection can be a treatment option for haemoptysis. If all PVs are stenotic and resection is not possible, patching can be performed under cardiopulmonary support.^4^ In our case, the irreversible vascular changes led to reduced pulmonary blood flow, and pulmonary resection was performed which resulted in control of haemoptysis and improvement of respiratory status.

Currently, there are no guidelines for follow-up after ablation, but there is a report that it is desirable to perform imaging with CT or magnetic resonance imaging at 3-4 months is recommended.^5^ When PV stenosis occurs after ablation, the stenosis progresses gradually, and the longer the time between the occurrence of the stenosis and endovascular therapy, the less improvement in therapeutic efficacy has been reported.^3^ If severe PV stenosis persists over a long period of time, irreversible changes in the pulmonary artery and vein may occur, as in our case, and pulmonary resection may become necessary. Therefore, long-term imaging follow-up is important, though further case accumulation is needed to determine the optimal timing. At the very least, even in asymptomatic patients, CT should be considered at 3 and 6 months post-ablation, and promptly performed if symptoms develop.

In conclusion, if haemoptysis occurs long after PV stenosis after ablation, irreversible changes in PV may have occurred, and anatomical lung resection may be a treatment option.

## Data Availability

Data are available in a repository and can be accessed using a unique identifier other than a DOI.
